# PlantEFRSegnet: A Plant Point Cloud Segmentation Network Based on Edge Point Preservation and Feature Feedback Repair

**DOI:** 10.3390/s26103104

**Published:** 2026-05-14

**Authors:** Bin Li, Peng Liu, Yonghan Zhang

**Affiliations:** 1 School of Computer Science, Northeast Electric Power University, Jilin 132012, China; 2 School of Mathematical and Computer Sciences, Heriot-Watt University, Edinburgh EH14 4AS, UK

**Keywords:** plant phenotyping, plant point cloud, point cloud downsampling, point cloud segmentation

## Abstract

The segmentation of 3D point clouds of plant organs, such as leaves and stems, helps to monitor plant growth and is a key step in plant growth phenotype analysis. Compared to point cloud segmentation tasks in other fields, plant point cloud segmentation is more challenging due to the interwoven distribution of various parts such as stems, leaves, and flowers. In this paper, we propose a universal point cloud segmentation network PlantEFRSegnet that can be used for multi-species of plants. The proposed PlantEFRSegnet utilizes a newly designed edge point preservation downsampling module to identify and preserve the points at the edges of plant organs during the downsampling process, in order to assist the segmentation network in learning the contours of various plant organs. PlantEFRSegnet performs supervised feature repair on the point cloud features obtained through downsampling to mitigate the impact of feature loss on segmentation performance during feature embedding. The encoder of the segmentation network is composed of four local feature extraction modules. These four modules can not only extract features but also enhance the features corresponding to points with high contributions in local regions based on point attention mechanism. We evaluated the proposed PlantEFRSegnet on a laser-scanned plant point cloud dataset. Compared with the state-of-the-art approaches, the proposed PlantEFRSegnet achieved better segmentation results.

## 1. Introduction

Plant point clouds play a crucial role in the study of crop morphology and structure due to their accurate and multi-angle representation capabilities. Plant point cloud segmentation aims to separate the point cloud structures of plant organs such as stems, leaves, and flowers, laying the foundation for further evaluation of plant traits and characteristics [1]. Accurately segmenting plant organs from disordered, unstructured, and densely uneven point clouds represents a current research focus and a challenging task.

Two-dimensional images have also been extensively used for extracting plant phenotypes. Angelo Cardellicchio et al. [2] successfully achieved the precise identification of phenotypic features such as nodes, fruits, and flowers in tomato plant images using YOLOv5. Yang et al. [3] proposed an improved YOLOv7 model to address the challenges of high density, overlap, and occlusion in apple fruit recognition. This model significantly improves the recognition accuracy and recall rate of YOLOv7 for apple fruits in complex environments by integrating attention modules and adding strategies such as auxiliary detection heads. The method of plant phenotype extraction based on two-dimensional images can partially solve the problems of low efficiency and subjectivity of traditional artificial phenotype methods. However, two-dimensional plant images do not reflect the true spatial morphology of plants, and there are phenomena such as occlusion and overlap, which can lead to measurement errors and other issues.

Advancements in point cloud acquisition devices and deep learning technologies have enabled the widespread application of 3D point cloud-based deep learning methods for plant organ segmentation and phenotype extraction tasks. Liu et al. [4] proposed PointNetPlus for cotton plant point cloud segmentation. Similarly, 3D point cloud-based deep learning methods have been applied to plant point cloud segmentation tasks for various specialized crops, including maize [5,6], tomato [7,8], rapeseed [9,10], and others [11,12,13,14,15,16,17]. These methods have been successfully implemented in specific plant point cloud segmentation tasks. But there are still two challenging issues that have not been resolved. First, these deep learning networks are often tailored for a single crop. These networks lack universality in crop variety segmentation tasks with different leaf shapes and canopy structures, which limits their widespread application. Thus, designing a universal 3D segmentation method for diverse crops represents a current frontier in plant 3D phenotyping research. Second, unlike general objects with distinct boundaries, the point clouds of organs such as stems, leaves, and flowers of plants are interwoven with each other. Therefore, it is necessary to design a dedicated plant feature extraction downsampling module. In addition, the field of plant point cloud segmentation still lacks an open-source annotated dataset containing multi-species of plant point clouds for fair comparison among various methods. The plant point cloud dataset established by Conn et al. [18,19] is widely used by researchers to evaluate segmentation methods. However, this dataset does not provide semantic annotations.

At present, there are few universal plant point cloud segmentation networks that can be applied to multiple plant species. This is because specialized segmentation networks can design dedicated feature extraction methods based on the characteristics of the plant species being segmented. For universal plant segmentation networks, the features of the objects being segmented vary greatly, making it difficult to improve segmentation quality through any specific point cloud feature extraction method. To address this issue, Li et al. proposed PlantNet [20]. PlantNet employs an edge-preservation strategy that effectively mitigates the loss of edge points during downsampling, thereby enhancing segmentation network performance. PSegNet [21] utilizes a voxel-based farthest-point downsampling method to keep the critical details of the original plant point cloud as much as possible. Li et al. [22] proposes a self-organizing map-based downsampling strategy specifically tailored for plant point clouds. The proposed method performs shape-aware sampling on irregular plant point clouds through preliminary semantic autoencoding of different organ types. Experiments on multi-class plant datasets demonstrate that this subsampling approach enhances the accuracy of downstream segmentation networks. These studies indicate that incorporating effective subsampling methods upstream of segmentation networks not only reduces computational load but also preserves edge details and other segmentation-enhancing features.

Existing plant point cloud segmentation networks are mostly derived from the modifications of point cloud segmentation networks that segment general objects. PointNet [23] and PointNet++ [24] are two typical point cloud segmentation networks that are universal for various objects. These two networks were the first semantic segmentation architectures to directly operate on unordered point clouds. Following the introduction of PointNet and PointNet++, improvements in PointNet-like frameworks primarily focused on enhancing network performance through the addition of residual connections or redesigning feature extraction modules. Yang et al. [5] proposed HAIS-PointNet for corn stalk and leaf point cloud segmentation. Yao et al. [7] proposed the CAFPoint network for tomato point cloud segmentation by incorporating a Cross Attention Fusion module into PointNet. Du et al. [9] incorporated the ASAP attention module into PointNet and proposed ASAP-PointNet. The ASAP-PointNet demonstrated strong semantic segmentation performance on the Chinese cabbage point cloud dataset. However, due to the interweaving of point clouds of plant organs such as stems, leaves, and flowers, network frameworks designed for general object segmentation tasks struggle to fully adapt to plant point cloud segmentation. To address the reduced segmentation accuracy caused by organ intertwining, Li et al. [25] abandoned the PointNet-like architecture and designed a dual-branch network incorporating global and local feature extraction. This network employs transformer self-attention and multi-head attention mechanisms to extract point cloud features, and designed a point cloud segmentation network for rose plant segmentation. Li et al. [25] points out that the stems and leaves of plants are interwoven together, and segmentation networks often extract point cloud features of the entire plant indiscriminately, without mining the relationships between point clouds belonging to the same organ in the point cloud space.

To address two challenge issues in plant segmentation tasks, we propose PlantEFRSegnet—a multi-species plant point cloud segmentation network. The edge point preservation (EPP) downsampling module of PlantEFRSegnet is responsible for downsampling plant point clouds. The EPP module maps point clouds onto an PCA plane and determines whether each point is an edge point in polar coordinates. The EPP module mixes edge points with interior points, then continuously performs supervised feature restoration [26] within PlantEFRSegnet’s segmentation network. EPP’s polar-coordinate-based edge point determination method avoids the difficulty of specifying edge point angle parameters encountered in PlantNet [20]. The downsampled point cloud undergoes supervised feature repair within the segmentation network to mitigate information loss during feature embedding. The point cloud segmentation network of PlantEFRSegnet consists of an encoder consisting of four local feature extraction (LFE) modules. These LFE modules enhance the features of high contribution points in local regions through a point attention mechanism. The contributions of this paper are as follows:

1. An edge point preservation (EPP) downsampling module was proposed. This module possesses high edge shape perception capabilities, making it highly suitable for downsampling tasks involving plant point clouds with irregular edges.
2. A universal plant point segmentation network PlantEFRSegnet was proposed. The proposed PlantEFRSegnet first uses the EPP module to extract the edge points, and then mixes the edge points with the internal points, and uses the segmentation network containing the feature feedback repair (FFR) module and LFE module for segmentation.
3. We annotated the plant point cloud dataset established by Conn et al. [18,19] and made it available for open-source download. We evaluated the proposed PlantEFRSegnet on this plant point cloud dataset and demonstrated its effectiveness.
4. The rest of the paper is arranged as follows. The proposed PlantEFRSegnet is described in detail in Section 2. Section 3 presents comparative experiments and ablation studies to demonstrate the effectiveness of the proposed PlantEFRSegnet. The conclusion is drawn in the last section.

## 2. Methods

**Figure 1:**
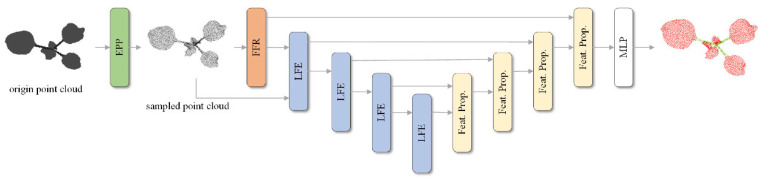
Architecture of PlantEFRSegnet.

The architecture of PlantEFRSegnet is shown in Figure 1. The proposed PlantEFRSegnet first employs the edge point preservation (EPP) downsampling module to perform edge-preserving downsampling on the input plant point cloud to be segmented, yielding a downsampled point cloud containing a mixture of contour edge points and interior points. Subsequently, PlantEFRSegnet utilizes the feature feedback repair (FFR) module to perform supervised repair on the downsampled point cloud. The main segmentation network of PlantEFRSegnet adopts an encoder–decoder architecture. The encoder contains four local feature extraction (LFE) modules. The decoder employs the same four feature propagation modules identical to those in [24]. The network concludes with a multi-layer perceptron (MLP).

### 2.1. Edge Point Preservation Downsampling

**Figure 2:**
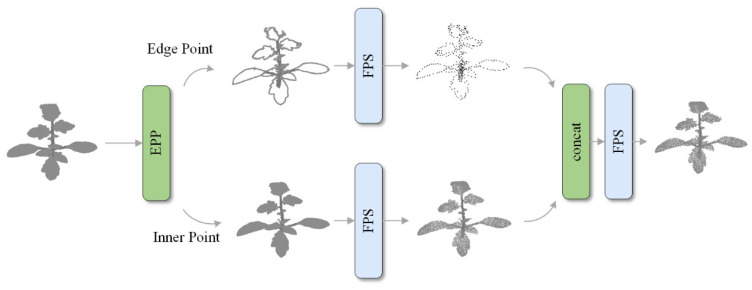
Edge point preservation downsampling method.

We propose an edge point determination method. As shown in Figure 2, during the downsampling process, this method is first used to filter out edge points and perform FPS downsampling on the edge points and internal points. The edge points and inner points obtained by downsampling are mixed in a ratio of 1:9. By filtering the edge points, the downsampling process can focus on preserving the edge points of plant organs to help the segmentation network learn the contours of plant organs.

**Figure 3:**
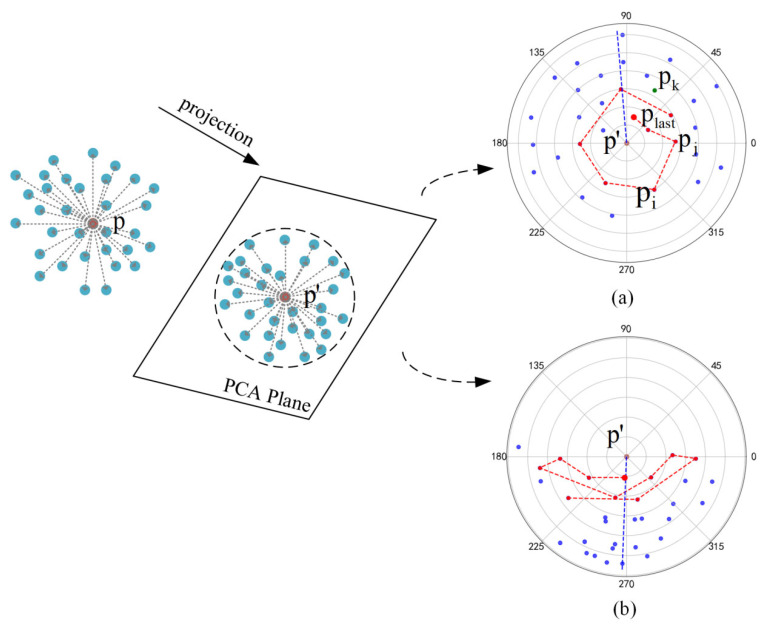
Process diagram of edge point detection method.

As shown in Figure 3, the proposed edge point determination method projects the current point p and its k nearest neighbors onto a polar coordinate plane. Let $p^{\prime }$ denote the projection point of p. Subsequently, the method searches for a path encircling point $p^{\prime }$. If a path encircling $p^{\prime }$ is found (Figure 3a), the corresponding point p is classified as an interior point. If no path encircling $p^{\prime }$ is found (Figure 3b), p is identified as an edge point.

The main steps of the edge point determination method are shown in Figure 3 and are as follows: (1) We calculate the principal component analysis (PCA) plane for point p and its k nearest neighbors. (2) To ensure the projected point of p can be in the center of the plane, we project the vectors of point p and its k nearest neighbors onto the PCA plane. (3) We transform the coordinate system containing the projected point $p^{\prime }$ and its k nearest neighbors into polar coordinates. (4) We calculate the average polar angle of all k nearest neighbors of $p^{\prime }$ in the polar coordinate system. To mitigate the influence of trigonometric periodicity on the average angle value, the method in [27] is used to calculate the average polar angle of the k nearest neighbor of $p^{\prime }$, as shown in Equations (1)–(3).

(1) $$\begin{array}{c} \theta_{m}=arctan2\left({sin}_{sum},{cos}_{sum}\right)\%360^{\circ } \end{array}$$

(2) $$\begin{array}{c} {sin}_{sum}=\sin\left(\theta_{1}\right)+\sin\left(\theta_{2}\right)+\cdots +\sin\left(\theta_{k}\right) \end{array}$$

(3) $$\begin{array}{c} {cos}_{sum}=\cos\left(\theta_{1}\right)+\cos\left(\theta_{2}\right)+\cdots +\cos\left(\theta_{k}\right) \end{array}$$

where $\theta_{1},\theta_{2},\cdots ,\theta_{k}$ denote the polar angles of each point. We assume the dashed lines in Figure 3a,b represent the calculated average polar angles.

(5) We use Formula (4) to normalize the polar diameter of all neighboring points of $p^{\prime }$. Here, $r_{j}$ denotes the polar diameter of the jth point in the polar coordinate system.

(4) $$\begin{array}{c} r_{j}=\frac{r_{j}-r_{min}}{r_{max}-r_{min}} \end{array}$$

(6) We search for a path encircling point $p^{\prime }$. First, we use Formulas (5)–(7) to find the point closest to $p^{\prime }$, and we use it as the starting point of the encircling path. This approach ensures the path tightly encircles point $p^{\prime }$. Inspired by [28], we compute a parameter $\gamma_{j}$ for each neighbor of $p^{\prime }$ and select the point with the smallest $\gamma_{j}$ value as the starting point. Since $p^{\prime }$ is the center of the polar coordinate system, Formula (5) selects the point where the difference between its polar angle and average angle is the smallest and its polar diameter is the shortest as the starting point. Such a point is closest to the center of the polar coordinate system, that is, closest to $p^{\prime }$. Let us suppose that the starting point is indicated by the largest red dot in Figure 3a,b.

(5) $$\begin{array}{c} \gamma_{j}=r_{j}+\left|\frac{\theta_{sub}}{360^{\circ }}\right| \end{array}$$

(6) $$\begin{array}{c} \theta_{sub}=\left(\theta_{j}-\theta_{m}\right)\%360^{\circ } \end{array}$$

(7) $$\begin{array}{c} \theta_{sub}=\left\{\begin{array}{c} \theta_{sub}-360^{\circ }\;if\;\theta_{sub}>180^{\circ }\\ \theta_{sub}\;otherwise \end{array}\right. \end{array}$$

**Figure 4:**
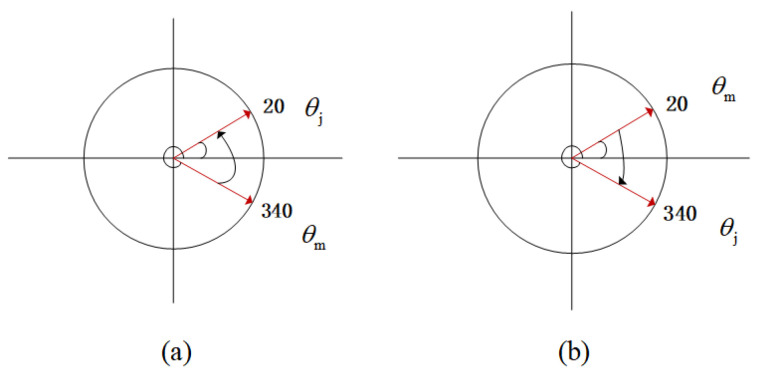
Difference between two angles.

The method in [28] is used to find edge points on point clouds with uniform density. This method is used for the edge of turbine blades in a turbofan engine. We have modified the normalization logic of the relative angle difference based on its method. Our method also adds a large-angle suppression factor to make it suitable for plant point clouds with uneven sparsity. The angular value is a number ranging from $0^{\circ }$ to $360^{\circ }$. In addition to representing the difference between the two angles, $\theta_{j}-\theta_{m}$ also represents the angle rotated from the polar diameter with angle $\theta_{m}$ to the polar diameter with angle $\theta_{j}$, in the counterclockwise direction (Figure 4a) or clockwise direction (Figure 4b). Equations (6) and (7) can ensure that $\theta_{sub}\in [{-180}^{\circ },180^{\circ }]$.

Starting from the starting point (the largest red dot in Figure 3a), we select a point $p_{j}$ from the nearest neighbor of $p^{\prime }$ each time and add it to the encircling path of $p^{\prime }$. For each nearest neighbor $p_{j}$ of $p^{\prime }$, we calculate the parameter $\varepsilon_{j}$ and select the point with the smallest $\varepsilon_{j}$ value to add to the encircling path. The formula is as follows:

(8) $$\begin{array}{c} \varepsilon_{j}=r_{j}+\left[\left|\frac{\theta_{j}-\theta_{last}}{360^{\circ }}\right|\cdot c_{1}\right]+c_{2} \end{array}$$

where $\theta_{last}$ represents the polar angle of point $p_{last}$ added to the encircling path in the previous round. $c_{1}$ is the direction retention factor with an absolute value greater than 1. Let us suppose that, in the previous round, the $p_{last}$ was added at the end of the clockwise growing encircling path. In this round, if $p_{j}$ is in the clockwise direction of $p_{last}$, the value of $c_{1}$ is negative; otherwise, it is positive. Conversely, if $p_{last}$ from the previous round was added at the end of a counterclockwise growing encircling path, then in this round, if $p_{j}$ lies counterclockwise of $p_{last}$, the value of $c_{1}$ is negative; otherwise, it is positive. As shown in Figure 3a, $p_{k}$ (the green point in Figure 3a) is in the counterclockwise direction of $p_{last}$, while $p_{j}$ is in the clockwise direction of $p_{last}$. The c_1_ values of $p_{k}$ and $p_{j}$ are positive and negative, respectively. The corresponding $\varepsilon_{j}$ value for $p_{j}$ may be smaller, making the probability of selecting $p_{j}$ greater than that of $p_{k}$. $c_{2}$ is a large-angle suppression factor with an absolute value greater than 1. If the angular difference between $p_{j}$ and $p_{last}$ exceeds 90 degrees, $c_{2}$ is positive; otherwise, it is zero. As shown in Figure 3a, the angular difference between $p_{i}$ and $p_{last}$ is greater than 90 degrees, while the angular difference between $p_{j}$ and $p_{last}$ is less than 90 degrees, the probability of $p_{j}$ being selected in this round is greater than $p_{i}$.

Formulas (9) and (10) define the angular increment α and the absolute value of the angular increment β, respectively, during the process of finding the encircling path.

(9) $$\begin{array}{c} \alpha =\alpha +\left(\theta_{j}-\theta_{last}\right) \end{array}$$

(10) $$\begin{array}{c} \beta =\beta +\left|\theta_{j}-\theta_{last}\right| \end{array}$$

In the iterative process of generating the surrounding path, real-time detection of the path’s coverage completeness is crucial for determining the properties of the center point. This paper quantifies the degree of path encirclement by accumulating angular increments. Specifically, after each new node on the surrounding path is determined, the algorithm calculates the angular increment α and the absolute value of the angular increment β in real time. When the absolute value of the angular increment β reaches or exceeds 360 degrees, it indicates that if the path continues to evolve in the same encircling direction, its topological trajectory is sufficient to achieve full coverage of the projected center point p′. At this point, to optimize computational efficiency, the algorithm triggers an early stopping strategy, ceasing the search for subsequent neighboring points. After the search stops, the algorithm performs the final determination by checking whether the absolute value of angular increment α crosses the threshold of 360 degrees. If $\alpha \geq 360^{\circ }$, it indicates that is a path that can enclose the point p′ being tested, and p′ is an interior point; otherwise, there is no path that can enclose the point p′, and p′ is an edge point. This early stopping strategy avoids redundant calculations and significantly improves the computational efficiency of the plant point cloud processing.

The whole process of the proposed edge point determination algorithm is explained in Algorithm 1.
**Algorithm 1 Edge point determination algorithm**$\mathrm{Input}:\;\mathrm{The}\;\mathrm{set}\;E\;\mathrm{of}\;k\;\mathrm{nearest}\;\mathrm{neighbors}\;\mathrm{of}\;p^{\prime }$, α, β$,\;\mathrm{path}\;(\mathrm{The}\;\mathrm{set}\;\mathrm{of}\;\mathrm{points}\;\mathrm{on}\;\mathrm{the}\;\mathrm{path}\;\mathrm{encircling}\;\mathrm{point}\;p^{\prime }$)**Output:** Is p an edge point  1: **for** p in E **do**  2:  
$\mathrm{Use}\;\mathrm{Formulas}\;(5)–(7)\;\mathrm{to}\;\mathrm{calculate}\;\gamma_{j}$
  3: **end for**  4: $\mathrm{Find}\;\mathrm{the}\;\mathrm{point}\;p_{j}$$\;\mathrm{corresponding}\;\mathrm{to}\;\mathrm{the}\;\mathrm{smallest}\;\gamma_{j}$$\;\mathrm{as}\;\mathrm{the}\;\mathrm{starting}\;\mathrm{point},\;\mathrm{and}\;\mathrm{add}\;p_{j}$ to the path.  5: E ⇐E − p  6: 
$\theta_{last}=\theta_{j}$
  7: **while** E is not empty **do**  8:  **for** p in E **do**
  9:   
$\mathrm{Use}\;\mathrm{Formulas}\;(6)–(8)\;\mathrm{to}\;\mathrm{calculate}\;\varepsilon_{j}$
   10:  **end for**   11:  $\mathrm{Find}\;\mathrm{the}\;\mathrm{point}\;\mathrm{corresponding}\;\mathrm{to}\;\mathrm{the}\;\mathrm{smallest}\;\varepsilon_{j}$, and add it to the path   12:  E ⇐E − p   13:  
$\alpha =\alpha +\left(\theta_{j}-\theta_{last}\right)$
   14:  
$\beta =\beta +|\theta_{j}-\theta_{last}|$
   15:  
$\theta_{last}=\theta_{j}$
   16:  if β$>360^{\circ }$ **then**   17:   break   18:  **end if**   19: **end while**   20: if |α$|>360^{\circ }$ **then**   21:  $\mathrm{There}\;\mathrm{exists}\;a\;\mathrm{path}\;\mathrm{encircling}\;p^{\prime }$$,\;\mathrm{where}\;p^{\prime }$ is an interior point, and the corresponding point p is also an interior point.   22: **else**   23:  $p^{\prime }$ is an edge point; the corresponding point p is an edge point.   24: **end if**

### 2.2. Feature Feedback Repair Module

Feature embedding is crucial for feature learning, and low-loss feature representations form the foundation for subsequent learning tasks in the network. Traditional feature embedding methods typically employ convolutional layers to accomplish this, inevitably leading to insufficient feature extraction or loss of original information. In our proposed segmentation network, we utilize the feature restoration method introduced by Zhao et al. [26] to perform supervised restoration on downsampled point clouds. The primary function of the FFR module is to map the downsampled point cloud $F\subseteq R^{3+H}$ into the embedded feature space $F_{embed}\subseteq R^{D}$; where 3 represents the three-dimensional coordinates of the point cloud; H denotes the height difference in the point cloud, i.e., the height of each point minus the minimum height; D represents the dimension of the feature space; $F_{embed}$ denotes the input of the segmentation network.

For any input point $p_{i}$, we compute the feature corresponding to its most salient edge and add the feature to the set $F_{forward}$. First, we find the k neighbors $p_{j}$ of $p_{i}$ by the KNN algorithm. Let the feature corresponding to $p_{i}$ be $f_{i}$, and the feature corresponding to $p_{j}$ be $f_{j}$. We use Formula (11) to compute the edge feature $e_{ij}$ between $p_{i}$ and its k neighbors $p_{j}$. Then, we use Formula (12) to select the largest edge feature in this round and add it to the set $F_{forward}$.

(11) $$\begin{array}{c} e_{ij}=ReLU\left(h_{1}\left(f_{j}-f_{i}\right),h_{1}\left(f_{i}\right)\right) \end{array}$$

(12) $$\begin{array}{c} {f^{\prime }}_{i}=\max\;e_{ij}\;\mathrm{where}\;j\in k \end{array}$$

(13) $$\begin{array}{c} F_{forward}=\left\{{f^{\prime }}_{1},{f^{\prime }}_{2},\ldots ,{f^{\prime }}_{n}\right\} \end{array}$$

where $h_{1}$ represents a multi-layer perceptron that increases the feature dimension.

Formula (14) performs a backward projection of $F_{forward}$ into the input features.

(14) $$\begin{array}{c} F_{feedback}=h_{2}\left(F_{forward}\right) \end{array}$$

where $F_{feedback}$ denotes features obtained through inverse projection, while $h_{2}$ represents a multi-layer perceptron that reduces feature dimensions. Formulas (15) and (16) compute the difference between $F_{feedback}$ and the point cloud F input to the FFR module, yielding the feature-restored point cloud feature $F_{embed}$.

(15) $$\begin{array}{c} w=softmax\left(h1\left(F-F_{feedback}\right)\right) \end{array}$$

(16) $$\begin{array}{c} F_{embed}=F_{forward}*w+F_{forward} \end{array}$$

where w denotes the learnable attention weights.

### 2.3. Local Feature Extraction Module

**Figure 5:**
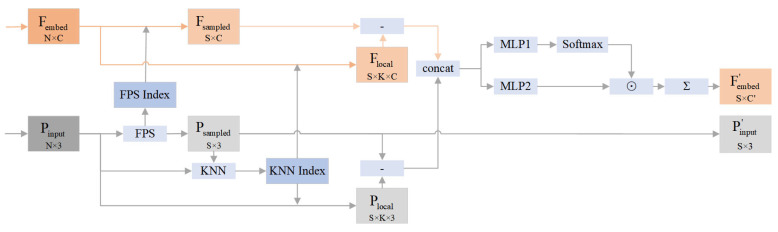
Structure of the local feature extraction module.

The local feature extraction (LFE) module of PlantEFRSegnet calculates the differences between local center points and neighborhood points, enhancing the features of points with significant contributions within local regions through a point attention mechanism. The structure of the LFE module is shown in Figure 5. The input of LFE module is the repaired feature $F_{embed}$ output by the FFR module and the plant point cloud $P_{input}$ downsampled by the EPP module. First, the LFE module performs FPS on $P_{input}$, and obtains the ${FPS}_{index}$ and the sampled point set $P_{sampled}$. Taking the point in $P_{sampled}$ as the central point, the k nearest neighbor (KNN) of each central point is calculated in $P_{input}$, and then ${KNN}_{index}$ is obtained. $F_{sampled}$ is obtained by retrieving $F_{embed}$ using ${FPS}_{index}$. Using ${KNN}_{index}$ to retrieve $F_{embed}$ and $P_{input}$, respectively, $F_{local}$ and $P_{local}$ were obtained. The difference between $F_{sampled}$ and $F_{local}$ represents the feature difference between local center points and neighborhood points. The difference between $P_{sampled}$ and $P_{local}$ represents the difference between the local center point and its neighborhood points. We concatenate the local feature difference and the point difference. The concatenate features are processed by a single-layer MLP1 and a three-layer MLP2, respectively. MLP1 and the Softmax function jointly determine the contribution of points within the neighborhood. Multiplying the Softmax function’s output by the features extracted by MLP2 achieves the goal of feature enhancement for points with significant feature contributions. $F_{embed}^{\prime }$ and $P_{input}^{\prime }$ are the inputs of the next LFE module.

### 2.4. Loss Function

The leaf area of plants is significantly larger than that of other organs. Therefore, the number of points corresponding to leaves are much higher than that of other categories. In general, the segmented network can easily classify the large proportion of leaf point cloud correctly. During training, the loss of these easily classified point clouds becomes dominant, overwhelming the loss of really difficult-to-classify point clouds and hindering effective model learning. We address this issue through the use of Focal Loss and Poly Loss.

The definition of Focal Loss [29] is:

(17) $$\begin{array}{c} FL\left(p_{t}\right)=-\alpha_{t}{\left(1-p_{t}\right)}^{\gamma }log\left(p_{t}\right) \end{array}$$

where $p_{t}$ is defined as follows:

(18) $$\begin{array}{c} p_{t}=\left\{\begin{array}{c} \;p\;\mathrm{if}\;y=1\\ 1-p\;\mathrm{if}\;y=0 \end{array}\right. \end{array}$$

p∈[0,1] represents the model’s predicted probability for the label y. $\alpha_{t}$ is the class balance factor. According to [29], when y = 1, $\alpha_{t}$ is set to 0.25; when y = 0, $\alpha_{t}$ is set to 0.75. During training, if a sample’s label y = 0, its predicted probability p is a value close to 1, that is, the network predicts the category of the sample incorrectly, and this is a difficult sample to classify. At this point, $\alpha_{t}$ and $p_{t}$ impose a higher penalty on this sample. γ is the focusing parameter. According to [29], the best results are achieved when γ = 2.

The definition of Poly Loss [30] is:

(19) $$\begin{array}{c} Poly\left(p_{t}\right)={\left(1-p_{t}\right)}^{\gamma +1} \end{array}$$

Poly Loss can impose a significant penalty on samples that are difficult to classify.

The proposed loss function for PlantEFRSegnet is:

(20) $$\begin{array}{c} Loss\left(p_{t}\right)=FL\left(p_{t}\right)+Poly\left(p_{t}\right)=-\alpha_{t}{\left(1-p_{t}\right)}^{\gamma }log\left(p_{t}\right)+\epsilon \cdot {\left(1-p_{t}\right)}^{\gamma +1} \end{array}$$

## 3. Experiment

### 3.1. Dataset

**Figure 6:**
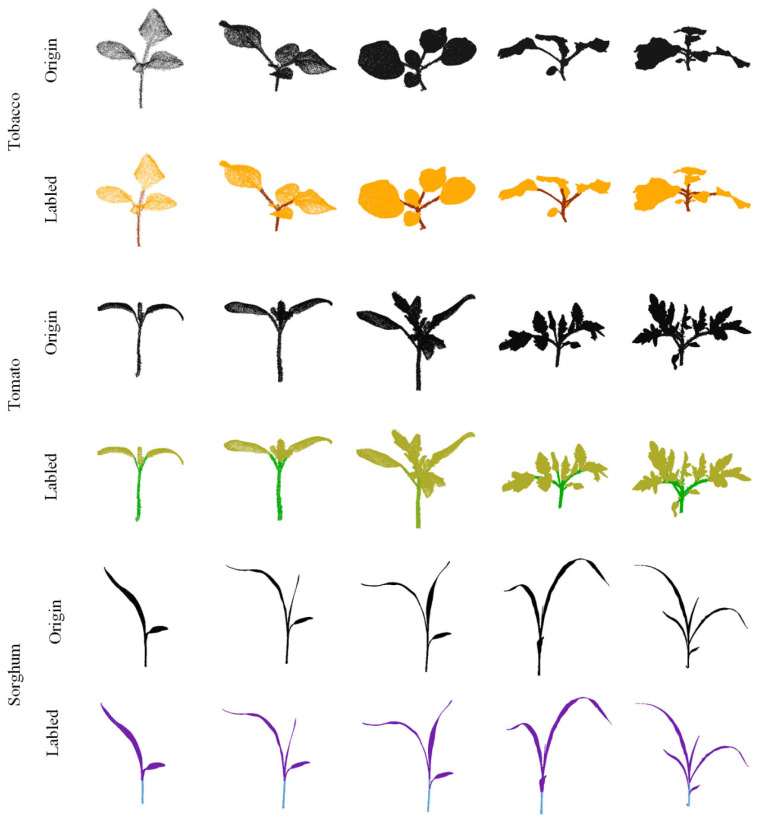
Visualization of plant point cloud and annotation point cloud.

A well-labeled plant point cloud dataset can provide a fair comparison platform for plant point cloud segmentation methods. We evaluated the performance of the proposed PlantEFRSegnet on the plant point cloud dataset released by Conn et al. [18,19]. This dataset includes three plant species: tobacco, tomato, and sorghum. High-resolution 3D laser scans were performed for each species under three to five growth conditions over a 30-day cycle. The dataset comprises 546 plant point cloud samples: 105 tobacco, 312 tomato, and 129 sorghum. These three species exhibit significant structural and leaf morphological differences. Tobacco and tomato are dicotyledons. Tobacco features an erect stem with sparse branching and large leaves arranged alternately. Tomato typically exhibits semi-trailing stems with irregular branching and densely arranged pinnate compound leaves. Sorghum, as a monocotyledonous plant, has an upright and robust stem and long, alternate leaves. Figure 6 displays selected samples from the dataset. As shown, the significant differences in stem and leaf morphology among these plants are suitable for evaluating universal plant point cloud segmentation networks.

We employed the point cloud annotation tool Semantic-Segmentation-Editor (SSE) to manually annotate the original point cloud dataset. Using this tool, we added semantic labels to the stems and leaves of each plant. The row corresponding to “origin” in Figure 6 is the original point cloud data in the plant point cloud dataset, while the row corresponding to “labeled” refers to the point cloud data formed after using semantic annotation tools for organ annotation.

### 3.2. Implementation Details

Before training, we applied the proposed edge point-preserving downsampling module to perform ten rounds of downsampling on the same plant’s point cloud, generating point clouds with subtle variations to augment the training data.

During training, this study employs the AdamW optimizer [31] with an initial learning rate of 0.001. A weight decay strategy is introduced, reducing the learning rate to 50% of its current value at iterations 150 and 210. The total training iterations amount to 300. Experiments were conducted on two NVIDIA T4 GPUs with a total batch size of 8. The system environment was Ubuntu 18.04, PyTorch 2.3.0 and CUDA 11.8. The number of input samples for the PlantEFRSegnet network is 4096. The decoder of PlantEFRSegnet has two layers, and the MLP of PlantEFRSegnet has three layers.

The relevant parameter settings of PlantEFRSegnet are shown in Table 1.

### 3.3. Evaluation Criteria

We employed four evaluation metrics to assess the performance of PlantEFRSegnet: precision, recall, F1 score, and intersection-over-union ratio (IoU). The specific definitions of these four evaluation metrics are as follows:

(21) $$\begin{array}{c} Precision=\frac{TP}{TP+FP} \end{array}$$

(22) $$\begin{array}{c} Recall=\frac{TP}{TP+FN} \end{array}$$

(23) $$\begin{array}{c} F1=2\cdot \frac{Precision\cdot Recall}{Precision+Recall} \end{array}$$

(24) $$\begin{array}{c} IoU=\frac{TP}{TP+FP+FN} \end{array}$$

where TP, FP, and FN are the number of true positive, false positive, and false negative points of a semantic class, respectively.

### 3.4. Segmentation Results

We selected nine popular methods for comparison with PlantEFRSegnet (PointNet [23], PointNet++ [24], ASIS [32], DGCNN [33], PlantNet [20], PSegNet [21], PointNeXt [34], SPoTr [35], PointCloudMamba [36]). Among the methods in Table 2, PlantEFRSegnet achieved leading performance on both tobacco and tomato plant categories. Only on sorghum, PlantEFRSegnet performs slightly worse than PointCloudMamba. PointCloudMamba is the latest method proposed in 2025. The consistency traversal serialization strategy adopted by this method coincides with the upright stem of sorghum plants. But, in other categories, PlantEFRSegnet performs better than PointCloudMamba. This indicates that our PlantEFRSegnet has better universality.

In the experiments, all methods achieved significantly higher segmentation accuracy for leaves than for stems. This primarily stems from two factors: first, leaves contain far more points than stems, especially during the later stages of plant growth. This higher point density provides models with richer feature information, making it easier for them to learn the morphological features of leaves. Second, leaves typically grow densely and exhibit a certain degree of spatial regularity in their arrangement. In contrast, the stems are slender in structure and interweave among the leaves in space, resulting in a sparse and occluded point cloud distribution, which increases the difficulty of segmentation. The proposed PlantEFRSegnet employs a loss function combining Focal Loss and Poly Loss, which partially addresses these challenges. Consequently, PlantEFRSegnet achieves superior segmentation performance on tobacco and tomato leaves compared to other methods.

In summary, the proposed PlantEFRSegnet outperforms state-of-the-art methods such as PointCloudMamba, SPoTr, and PointNeXt. Compared to the second-best method (PointCloudMamba), it achieves improvements of 0.52%, 0.27%, 0.40%, and 0.73% in average precision, average recall, average F1 score, and average IoU, respectively.

### 3.5. Visual Effects

**Figure 7:**
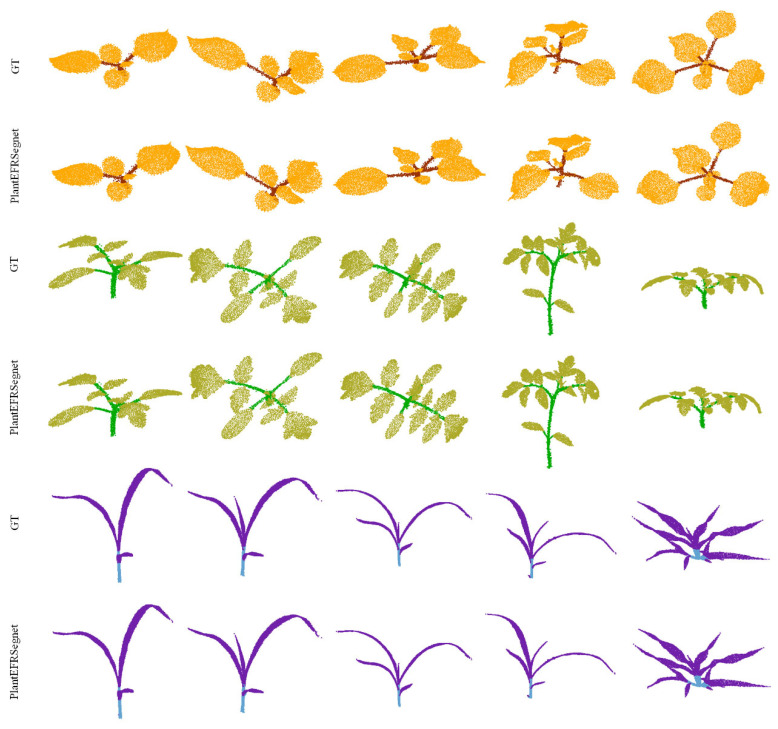
Visualization of PlantEPRSegnet’s semantic segmentation performance on three plant species.

Figure 7 displays the semantic segmentation results of PlantEPRSegnet for tobacco, tomato, and sorghum, from top to bottom. GT denotes the ground truth label for a plant point cloud. To simulate different growth stages and environments, we selected test samples from diverse growth conditions and phases. From Figure 7, we can see that PlantEPRSegnet performs well in segmenting all three crops. Compared with the other two crops, the canopy structure of tomato plants is more complex, and the number of leaves is denser. Their samples demonstrate significant spatial morphological diversity, with leaves exhibiting a wide range of curvature. Despite these challenges, PlantEPRSegnet achieves robust segmentation performance on tomato samples. In the sorghum subdataset, it is often difficult to accurately distinguish the connection area between leaves and stems, but PlantEFRSegnet’s segmentation performance in these areas are satisfactory.

**Figure 8:**
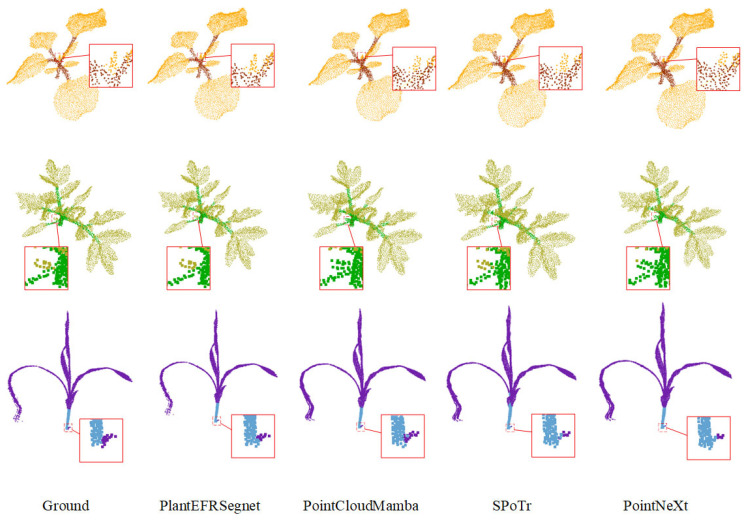
Qualitative semantic segmentation comparison of four methods.

We conducted a visual comparison analysis between PointCloudMamba, SPoTr, and PointNeXt against our PlantEFRSegnet. The semantic segmentation results for the three species are shown in Figure 8. As indicated by all the boxes of Figure 8, the segmentation effect of the proposed PlantEFRSegnet for key regions is the most close match of the ground truth (GT). Both PointCloudMamba and PointNeXt misclassified leaves as stems within the boxed regions. SPoTr misclassified a small portion of leaves as stems within the boxed regions. In sorghum plant segmentation, PlantEFRSegnet performed slightly worse than PointCloudMamba, which confirms the segmentation results in Table 2. However, PlantEFRSegnet outperformed PointCloudMamba in both tomato and sorghum segmentation tasks. This indicates that PlantEFRSegnet has better universality than PointCloudMamba.

### 3.6. Ablation Studies

In this section, we validated the effectiveness of each module in PlantEFRSegnet through multiple sets of ablation studies. The results are shown in Table 3. The checkmark in Table 3 indicates that the model contains a specific module. N1, N2, and N3 are networks that remove a certain module, and N4 represents the complete PlantEFRSegnet network. In the network without EPP module (N1 and N3), we use the FPS algorithm for downsampling. In the network without the FFP module (N1 and N2), we use MLP with the same depth as FFP to maintain consistency with the feature dimensions’ output by the original network. The experimental results show that the complete version of PlantEFRSegnet achieved the best results in various evaluation indicators such as average accuracy, recall, F1 score, and IoU, further proving the effectiveness of each module of PlantEFRSegnet.

From Table 3, it can be seen that the evaluation results of model N2 and model N3 are similar in some categories. For example, in the leaf of the sorghum category, model N2 and model N3 achieved recall values of 99.16 and 99.25 and F1 values of 99.23 and 99.29, respectively. This seems to prove that the FFR module is useless. However, models N2 and N3 achieved IoU values of 86.49 and 88.06, respectively, in the stem of the sorghum class. We believe that the generalization of the model is reflected in the segmentation of these difficult categories, so the FFR module is helpful for the overall performance of PlantEFRSeg.

## 4. Conclusions

In this paper, we propose a universal plant point cloud segmentation network PlantEFRSegnet. The proposed PlantEFRSegnet consists of an edge point preservation downsampling module and a segmentation network with a feature repair module. The proposed PlantEFRSegnet achieved semantic segmentation results of 97.47%, 97.28%, 97.30%, and 94.95% on the average precision, recall, F1 score, and IoU of tobacco, tomato, and sorghum categories, respectively. The performance of PlantEFRSegnet is superior to the compared state-of-the-art methods. From the visualization results, it can be seen that the segmentation performance of PlantEFRSegnet is more accurate and versatile in segmenting stems and leaves compared to the segmentation results of the comparative methods. The isolation experiment verified the effectiveness of the edge point preservation downsampling (EPP) module and feature feedback repair (FFR) module in PlantEFRSegnet. The proposed PlantEFRSegnet requires approximately three times the inference time of the standard FPS method due to the use of the EPP module. We tested a tobacco plant with 138,968 points, and the inference time using the standard FPS method was 3 s, while with the method proposed in this paper, it was 9 s. Therefore, research on making the model lightweight is our next research direction.

We use the term “universal” to describe the proposed PlantEFRSegnet for the following two reasons. Firstly, the proposed PlantEFRSegnet is not designed specifically for a single type of plant point cloud segmentation task, and we have demonstrated the effectiveness of PlantEFRSegnet on three types of plant point clouds. The term “universal” is used to distinguish it from point cloud segmentation studies designed specifically for certain plants. Secondly, we also aim to follow the conventions of similar studies [20,21]. However, since the evaluation was only conducted on three types of plant point clouds, it should be noted that using the term “universal” to describe the proposed PlantEFRSegnet does not mean that we have proven its universality for all plant point cloud segmentation tasks.

## Figures and Tables

**Table 1:** Hyperparameter settings.

**Hyperparameter**	**Value**	**Description**
$c_{1}$	3	Direction retention factor
$c_{2}$	5	Large-angle suppression factor
k	32	Number of nearest neighbors considered
$\alpha_{t}$	$\alpha_{t}$ = 0.25 for y = 1 $\alpha_{t}$ = 0.75 for y = 0	Class balance factor
γ	2	Focusing parameter
ϵ	1	Polynomial coefficient

**Table 2:** Quantitative comparison of segmentation effects of different networks.

	Method	Tobacco		Tomato		Sorghum		Mean
		Stem	Leaf	Stem	Leaf	Stem	Leaf	
Precision (%)	PointNet	77.15	94.02	93.99	96.71	77.87	95.37	89.19
	PointNet++	87.78	95.62	93.65	96.80	78.01	98.33	91.70
	ASIS	91.65	91.94	93.55	97.14	85.47	95.17	92.49
	DGCNN	90.55	96.42	95.24	97.86	83.95	97.37	93.57
	PlantNet	89.45	96.80	95.90	96.30	89.07	97.43	94.16
	PSegNet	92.71	96.76	96.36	97.98	89.54	98.04	95.23
	PointNeXt (C = 160)	95.12	98.62	96.66	98.38	89.50	98.86	96.19
	SPoTr	96.00	98.85	97.70	98.09	91.37	99.18	96.87
	PointCloudMamba	96.41	98.78	96.38	98.58	92.06	99.51	96.95
	PlantEFRSegnet	96.65	99.30	97.47	98.98	93.22	99.49	97.52
Recall (%)	PointNet	79.20	93.31	91.85	97.61	61.45	97.85	86.88
	PointNet++	90.83	94.05	92.45	97.33	78.66	98.27	91.93
	ASIS	83.85	96.11	92.87	95.51	81.65	97.88	91.31
	DGCNN	85.55	97.76	94.15	98.27	78.05	98.20	92.00
	PlantNet	86.12	92.97	95.24	98.23	86.06	98.07	92.78
	PSegNet	87.42	97.73	95.02	98.59	85.69	98.63	93.85
	PointNeXt (C = 160)	92.59	99.21	94.52	99.10	89.32	98.93	95.61
	SPoTr	93.70	99.32	93.69	99.38	92.28	99.06	96.24
	PointCloudMamba	93.73	99.41	95.29	99.03	95.42	99.17	97.01
	PlantEFRSegnet	94.93	99.58	96.44	99.33	95.23	99.25	97.46
F1 (%)	PointNet	78.16	93.66	92.91	97.16	68.69	96.59	87.86
	PointNet++	89.28	94.83	93.05	97.07	78.33	98.30	91.81
	ASIS	87.58	93.98	93.21	96.32	93.52	96.51	91.58
	DGCNN	87.98	97.09	94.69	98.07	80.89	97.78	92.75
	PlantNet	87.75	94.85	95.56	97.26	87.54	97.75	93.45
	PSegNet	89.99	97.24	95.68	98.29	87.57	98.33	94.52
	PointNeXt (C = 160)	93.69	98.91	95.50	98.73	88.84	98.89	95.76
	SPoTr	94.77	99.08	95.57	98.72	91.43	99.11	96.45
	PointCloudMamba	94.98	99.09	95.77	98.80	93.41	99.34	96.90
	PlantEFRSegnet	95.61	99.43	96.92	99.15	93.42	99.29	97.30
IoU (%)	PointNet	64.15	88.08	86.76	94.48	52.31	93.41	79.87
	PointNet++	80.63	90.17	87.00	94.30	64.38	96.65	85.52
	ASIS	77.91	88.64	87.29	92.90	71.70	93.25	85.28
	DGCNN	78.54	94.34	89.92	96.20	76.92	95.65	88.60
	PlantNet	78.17	90.20	91.51	94.66	77.84	95.59	88.00
	PSegNet	81.79	94.63	91.73	96.63	77.89	96.72	89.90
	PointNeXt (C = 160)	88.48	97.85	91.56	97.50	80.58	97.81	92.30
	SPoTr	90.26	98.18	91.70	97.49	84.60	98.25	93.41
	PointCloudMamba	90.62	98.21	92.06	97.63	88.15	98.68	94.22
	PlantEFRSegnet	91.78	98.87	94.14	98.34	88.06	98.61	94.97

**Table 3:** Ablation study of PlantEFRSeg.

		EPP	FFR	Tobacco		Tomato		Sorghum		Mean
				Stem	Leaf	Stem	Leaf	Stem	Leaf	
Precision (%)	N1			96.03	98.87	97.21	98.71	92.98	99.19	97.17
	N2	√		96.51	99.21	97.40	99.02	92.16	99.30	97.27
	N3		√	96.27	98.85	96.93	98.75	92.30	99.35	97.08
	N4	√	√	96.65	99.30	97.47	98.98	93.22	99.49	97.52
Recall (%)	N1			93.94	99.35	95.66	99.24	92.20	99.22	96.60
	N2	√		94.58	99.56	96.52	99.30	93.57	99.16	97.12
	N3		√	93.98	99.41	95.92	99.19	94.14	99.10	96.96
	N4	√	√	94.93	99.58	96.44	99.33	95.23	99.25	97.46
F1 (%)	N1			94.98	99.11	96.38	98.97	92.10	99.19	96.79
	N2	√		95.54	99.39	96.93	99.16	92.57	99.23	97.14
	N3		√	94.98	99.12	96.38	98.97	93.38	99.29	97.02
	N4	√	√	95.61	99.43	96.92	99.15	93.42	99.29	97.30
IoU (%)	N1			90.48	98.23	93.15	97.97	85.91	98.41	94.03
	N2	√		91.62	98.80	94.12	98.32	86.49	98.47	94.64
	N3		√	90.68	98.27	93.12	97.96	87.99	98.59	94.44
	N4	√	√	91.78	98.87	94.14	98.34	88.06	98.61	94.97

## Data Availability

The experimental dataset used in this paper can be obtained through the following link: https://github.com/dllab23/PlantPointCloud (accessed on 11 May 2026).

## References

[1] Akhtar M.S., Zafar Z., Nawaz R., Fraz M.M. (2024). Unlocking plant secrets: A systematic review of 3D imaging in plant phenotyping techniques. Comput. Electron. Agric..

[2] Cardellicchio A., Solimani F., Dimauro G., Petrozza A., Summerer S., Cellini F., Renò V. (2023). Detection of tomato plant phenotyping traits using YOLOv5-based single stage detectors. Comput. Electron. Agric..

[3] Yang H., Liu Y., Wang S., Qu H., Li N., Wu J., Yan Y., Zhang H., Wang J., Qiu J. (2023). Improved apple fruit target recognition method based on YOLOv7 model. Agriculture.

[4] Liu F.-Y., Geng H., Shang L.-Y., Si C.-J., Shen S.-Q. (2025). A cotton organ segmentation method with phenotypic measurements from a point cloud using a transformer. Plant Methods.

[5] Yang X., Miao T., Tian X., Wang D., Zhao J., Lin L., Zhu C., Yang T., Xu T. (2024). Maize stem–leaf segmentation framework based on deformable point clouds. ISPRS J. Photogramm. Remote Sens..

[6] Miao Y., Wang L., Peng C., Li H., Zhang M. (2024). Single plant segmentation and growth parameters measurement of maize seedling stage based on point cloud intensity. Smart Agric. Technol..

[7] Yao J., Gong Y., Xia Z., Nie P., Xu H., Zhang H., Chen Y., Li X., Li Z., Li Y. (2025). Facility of tomato plant organ segmentation and phenotypic trait extraction via deep learning. Comput. Electron. Agric..

[8] Liang X., Yu W., Qin L., Wang J., Jia P., Liu Q., Lei X., Yang M. (2025). Stem and Leaf Segmentation and Phenotypic Parameter Extraction of Tomato Seedlings Based on 3D Point. Agronomy.

[9] Du R., Ma Z., Xie P., He Y., Cen H. (2023). PST: Plant segmentation transformer for 3D point clouds of rapeseed plants at the podding stage. ISPRS J. Photogramm. Remote Sens..

[10] Sun B., Zain M., Zhang L., Han D., Sun C. (2025). Stem-Leaf Segmentation and Morphological Traits Extraction in Rapeseed Seedlings Using a Three-Dimensional Point Cloud. Agronomy.

[11] Jiang L., Li C., Fu L. (2025). Apple tree architectural trait phenotyping with organ-level instance segmentation from point cloud. Comput. Electron. Agric..

[12] Ma L., Kong L., Peng X., Wang K., Geng N. (2024). PSTNet: Transformer for aggregating neighborhood features in 3D point cloud semantic segmentation of eggplant plants. Sci. Hortic..

[13] Guo R., Xie J., Zhu J., Cheng R., Zhang Y., Zhang X., Gong X., Zhang R., Wang H., Meng F. (2023). Improved 3D point cloud segmentation for accurate phenotypic analysis of cabbage plants using deep learning and clustering algorithms. Comput. Electron. Agric..

[14] Deng Q., Zhao J., Li R., Liu G., Hu Y., Ye Z., Zhou G. (2024). A precise segmentation algorithm of pumpkin seedling point cloud stem based on CPHNet. Plants.

[15] Zarei A., Li B., Schnable J.C., Lyons E., Pauli D., Barnard K., Benes B. (2024). PlantSegNet: 3D point cloud instance segmentation of nearby plant organs with identical semantics. Comput. Electron. Agric..

[16] Shen J., Wu T., Zhao J., Wu Z., Huang Y., Gao P., Zhang L. (2024). Organ segmentation and phenotypic trait extraction of cotton seedling point clouds based on a 3D lightweight network. Agronomy.

[17] Zhang Y., Xie Y., Zhou J., Xu X., Miao M. (2024). Cucumber seedling segmentation network based on a multiview geometric graph encoder from 3D point clouds. Plant Phenomics.

[18] Conn A., Pedmale U.V., Chory J., Navlakha S. (2017). High-resolution laser scanning reveals plant architectures that reflect universal network design principles. Cell Syst..

[19] Conn A., Pedmale U.V., Chory J., Stevens C.F., Navlakha S. (2017). A statistical description of plant shoot architecture. Curr. Biol..

[20] Li D., Shi G., Li J., Chen Y., Zhang S., Xiang S., Jin S. (2022). PlantNet: A dual-function point cloud segmentation network for multiple plant species. ISPRS J. Photogramm. Remote Sens..

[21] Li D., Li J., Xiang S., Pan A. (2022). PSegNet: Simultaneous semantic and instance segmentation for point clouds of plants. Plant Phenomics.

[22] Li D., Zhou Z., Wei Y. (2024). Unsupervised shape-aware SOM down-sampling for plant point clouds. ISPRS J. Photogramm. Remote Sens..

[23] Qi C.R., Su H., Mo K., Guibas L.J. Pointnet: Deep learning on point sets for 3d classification and segmentation. Proceedings of the IEEE Conference on Computer Vision and Pattern Recognition.

[24] Qi C.R., Yi L., Su H., Guibas L.J. Pointnet++: Deep hierarchical feature learning on point sets in a metric space. Proceedings of the 31st International Conference on Neural Information Processing Systems.

[25] Li B., Guo C. (2022). MASPC_Transform: A plant point cloud segmentation network based on multi-head attention separation and position code. Sensors.

[26] Zhao J., Liu Y., Wu B. (2024). Multi-scale learnable key-channel attention network for point cloud classification and segmentation. Appl. Soft Comput..

[27] Mardia K.V., Jupp P.E. (2009). Directional Statistics.

[28] Mineo C., Pierce S.G., Summan R. (2019). Novel algorithms for 3D surface point cloud boundary detection and edge reconstruction. J. Comput. Des. Eng..

[29] Lin T.-Y., Goyal P., Girshick R., He K., Dollár P. Focal loss for dense object detection. Proceedings of the IEEE International Conference on Computer Vision.

[30] Leng Z., Tan M., Liu C., Cubuk E.D., Shi X., Cheng S., Anguelov D. (2022). Polyloss: A polynomial expansion perspective of classification loss functions. arXiv.

[31] Loshchilov I., Hutter F. (2017). Decoupled weight decay regularization. arXiv.

[32] Wang X., Liu S., Shen X., Shen C., Jia J. Associatively segmenting instances and semantics in point clouds. Proceedings of the 2019 IEEE/CVF Conference on Computer Vision and Pattern Recognition (CVPR).

[33] Wang Y., Sun Y., Liu Z., Sarma S.E., Bronstein M.M., Solomon J.M. (2019). Dynamic graph cnn for learning on point clouds. Acm Trans. Graph..

[34] Qian G., Li Y., Peng H., Mai J., Hammoud H., Elhoseiny M., Ghanem B. (2022). Pointnext: Revisiting pointnet++ with improved training and scaling strategies. Advances in Neural Information Processing Systems.

[35] Park J., Lee S., Kim S., Xiong Y., Kim H.J. Self-positioning point-based transformer for point cloud understanding. Proceedings of the IEEE/CVF Conference on Computer Vision and Pattern Recognition.

[36] Zhang T., Yuan H., Qi L., Zhang J., Zhou Q., Ji S., Yan S., Li X. Point cloud mamba: Point cloud learning via state space model. Proceedings of the AAAI Conference on Artificial Intelligence.

